# Neurolaw: A brief introduction

**Published:** 2015-01-05

**Authors:** Arian Petoft

**Affiliations:** Department of Public Law, School of Law and Political Sciences, Allameh Tabatabai University, Tehran, Iran

**Keywords:** Brain, Human Behavior, Law, Legal Decisions, Legal Rules, Neurolaw, Neuroscience

## Abstract

Neurolaw, as an interdisciplinary field which links the brain to law, facilitates the pathway to better understanding of human behavior in order to regulate it accurately through incorporating neuroscience achievements in legal studies. Since 1990’s, this emerging field, by study on human nervous system as a new dimension of legal phenomena, leads to a more precise explanation for human behavior to revise legal rules and decision-makings. This paper strives to bring about significantly a brief introduction to neurolaw so as to take effective steps toward exploring and expanding the scope of law and more thorough understanding of legal issues in the field at hand.

## Introduction

The evolution of scientific theories is reliant on the integrity of their propositions through a comprehensive approach covering all aspects of subjects;^1^ law is of no exception to this principle too. Unprecedentedly, legal effects and consequences are tied to neurological issues; hence an appeal to neuroscience would be inevitable for a better explanation for legal rules. Relationship between law and neuroscience; with brain lying in as their similar correlative factor, gives rise to neurolaw as an interdisciplinary field, offering more comprehensive, accurate approach to legal phenomena; that all put forward a more accurate evidence for legal process, and a fairer justice system; moreover, the expansion of both sciences is a matter of neurolaw.

There are plenty of cases in which neuroscientific data might be of significance to more accurately understand legal issues. This is why lots of neuroscientific evidences are increasingly reaching courts in a number of legal contexts in practice. Neurolaw would generate a better and wiser judicial, even legislative and executive system. Neuroscience achievements could change legal provisions, along with procedural law and customs, or even alter them radically to a new different one. Hence, this paper tries to give a brief introduction to neurolaw in order to take effective steps toward exploring and expanding the scope of the law and more thorough understanding of legal issues in the field at hand.

***Neurolaw: the intersection of neuroscience and law***

Scientists with many investigations on the human brain have learned a tremendous amount about how it works, how it malfunctions, and how it can be repaired or altered. This emerging neuroscience, namely the scientific study of the nervous system, has already revolutionized medical practices. Neuroscience as a branch of biology is currently an interdisciplinary science that collaborates with other fields.^2^ It also proved to be an immediate and powerful catalyst to understanding how the nervous system works and also exerts influence upon neurolaw.^3^ Neurolaw is an attempt to know the relationship between law and brain by taking into account neuroscience findings.^4^ In fact, neurolaw explores the effects of discoveries in neuroscience on legal rules.^5^

The most fundamental question among neuroscientists and lawyers is possibility of the relationship between law and neuroscience. Neuroscience is a natural science which based on experiment and indicative statements; while law is a humanities' science according to obligations, arising from the collective wisdom and abstract propositions. As more legal scientists believe, actually, law is a social phenomenon which has been formed by the social contract. Hence, law based on relative propositions, while neuroscience is on absolute ones. This leads our mind to real challenge that how it possible to propose and defend “neurolaw”? In fact, law is humans’ creative^6^ to regulate individuals’ conducts insecure and excellence society, Instead of natural community in which there is no law, no state, people do whatever they want and security minimalized.^7^ The ultimate goal of law is respecting to human dignity, in order to realization of humanity of a person and real justice; this purpose is achievable if we have better and more accurate rules in society, In other words, have a more fair legal system. Neuroscientific statements, with an open eye on neurological phenomenon, help law to have more accurate rules on this sense. More clearly, neurolaw shed light on justice way for law in its specific scientific area. For example, when legislators want to adopt a specific act, which related to punish offenders, or when judges want to decide about an accused, neuroscience achievements give precise glasses to lawyers, to have a more comprehensive view and consequently decide more equitable and fair legal decisions.

Drawing from neuroscience, neurolawyers try to understand human behavior, and will potentially shape future aspects of legal processes. Practically, they deliberate on human brain and nervous system image by medical technology scanning such as radiology, psychiatry, neurology, and clinical neuropsychology.^8^ With these new imaging techniques, researchers interested in the function of the human brain were presented with an unprecedented opportunity to examine the neurobiological correlates of human behaviors. Essentially, neuroimaging methods create visual brain delineation and the imaging specialist interprets it.^9^ Initially, neuroscience has been more exploited for Procedural law to stand criminal and civil liability complaint in court. Despite this pragmatic application of neuroscience, it has been applied to many legal subfields. Today, we are witnessing the development of neuroscientific considerations in various areas of law; such as Intellectual Property Law, Tort Law, Consumer Law, Health Law, Employment Law, Constitutional Law, and Criminal Law.^10^ Even neurolaw perforate to scope of other related sciences; such as psychiatry, sociology, political science, behavioral ecology and economics that mainly emphasize on criminology.^11^

Neuroscience has shed light of enquiry on how the brain and certain mental processes can work, and it follows understanding structure and function of the brain. It gives us an insight into the mental processes that underpin human behavior as the law is primarily concerned with regulating people’s behavior. It shapes an interdisciplinary science known as neurolaw. Because of huge differences among individuals’ brains,^12^ however, there is no direct mapping of mental function to specific areas of it.^13^ This is a fundamental challenge in the neurolaw. Neurolaw scientists attempt to expose neuroscience results to legal rule and system; thereby, revise legal standards, norms, and conducts to a more accurate one. More precisely, the novel neuroscientific approach toward legal rules and consequences brings about a more perfect and better realization of legal effects; hereby, mutates the rules governing them so that a farrier legal system can be followed up.

**Neurocriminology**

Neurocriminology is a sub-discipline of criminology that applies neuroscience techniques to probe the causes and cures of crime. Neurocriminology studies the makeup and composition of the brain and looks for correlations between characteristics of the brain and criminal behavior. The very rapid developments taking place in brain-imaging science are creating a new approach to our concepts of responsibility and retribution on the one hand, and understanding and mercy on the other.^14^ Neurocriminology is documenting structural and functional brain impairments not just in antisocial, violent, and psychopathic individuals, but also in spouse abusers and white collar criminals. Neurocriminologists are proposing a neurodevelopmental contribution to crime causation. By neurocriminology researches, it is clarified that the brain circuits found to be impaired in offenders parallel the brain circuits found to underlie moral decision-making in controls. Recent researches in neurocriminology, are outlining implications not just for the field of criminology, but also for concepts of legal and moral responsibility, free will, and punishment. To this end, the legal implications of brain research, free will and the neural bases of antisocial or criminal behavior are of central importance. Understanding responsibility, free will, and punishment and their relationship profound debate brewed in neurocriminology; if the neural circuitry underling legality is compromised in offenders, is it morally and legally wrong of us to punish prisoners as much as we do? The relationship between belief in free will and third-party punishment of criminal norm violations have been the subject of great debates among philosophers, criminologists, and neuroscientists.

Free will is the often unspoken centerpiece of the criminal law, which presumes humans are responsible agents, who are free to choose to comply with social norms or violate them. While many texts discussing the forensic implications of neuroscience refer to cases where brain damage such as that caused by an accident, a tumor, or surgical resection is related to alleged criminal behavior; this is the idea thoughts criminal, antisocial, sociopathic, or psychopathic behavior is linked to focal lesions of the brain.^15^ Today, by neurocriminology studies, (Legal Responsibility) is far away from its classical sense. Neurocriminologists by considering, pondering and interpreting brain-imaging, endeavor to prove Relative offenders responsibility. There are multiple neuroscientific documents that imply the truth of their claims. To test their hypotheses, neurocriminologists combined functional magnetic resonance imaging (fMRI) with a third-party punishment task, asking healthy subjects to estimate how much punishment a hypothetical offender deserved for a set of prototypical offenses ranging across severity of crime from property destruction and theft to rape and murder.^16^

***A reflection on two main kinds of research in neurolaw***

Neurolaw is a relatively new and highly-interdisciplinary field while the Decade of the Brain^17^ was first introduced to the health care and legal communities. The term neurolaw, among legal scholars, was first coined by Taylor et al.^18^ More effectively, He raised the issue of it, with his prominent scientific paper entitled “Neuropsychologists and Neurolawyers.”^18^ Taylor’s works during his career in academia^19^ are of considerable significance in the research area of neurolaw, chiefly legal practice. Neuroscience and the law have interacted over a long history. Since 1990, however, neuroscientists and neurolawyers have often argued about eventuality of spreading neurolaw. Also, lecturers at several scientific conferences held in the United States, United Kingdom, France and Canada address this subject frequently.^20^

*Practical researches*

Neurolaw practical researchers are emphasizing on civil and criminal responsibility litigation and its practical challenges such as documenting neuroscientific data as evidence in the court room or neurolitigation problems. There exists much of legal process literature in the neurolaw. “Neuroscience and Legal Responsibility”^21^ is one of the most leading recent works. It explores the field of legal responsibility by adopting a broadly compatibilist approach. The author argued that how neuroscience, psychology, and behavioral genetics should impact legal responsibility practices? This book mainly is challenging traditional conceptions of free will and legal liability. By a comparative analysis, among other works, “International Neurolaw: A Comparative Analysis”^22^ compares the different legal systems and strategies that they offer for dealing with neurolaw implications; accordingly it is so important to understand different legal approaches to how revise legal system by new neuroscience results. Moreover, “Neurolaw for Trial Lawyers,”^23^ “Law and Neuroscience: Current Legal Issues,”^24^ “Neuroscience in the Courtroom,”^25^ “A Primer on Criminal Law and Neuroscience,”^26^ are another useful works offering aid in solving existing problems in legal practice. These works emphasize Procedural law and practical legal rule inspired by neurolaw. Practical neurolaw is most related to neurolitigation, applying these new criminal Procedural law standards in courtroom by judges and lawyers.

According to what was discussed, the main issues which have been proposed in neurolaw practical researches are as follows: neurolitigation challenges, neuroscientific Instruments for proving or compurgation legal responsibility, neurocriminology in Procedural law, standing neurolitigation, neuro advocacy and attorney, neuroscience and judgment, brain injury rights to appeal.

*Theoretical researches*

On the other hand, in the theoretical approach we understand the brain, its functions in the conceptual value. Such an approach is of particular importance in accentuating impact of brain on behavior. By this, we are recognizing new rules regulating these behaviors in the legal system. As neuroscientific technologies contribute to understanding of the mind, applying neuroscience discoveries in legal proceedings has also increased. Cognitive neuroscientists interrogate complex relationship between the mind and the brain. They do it rather by using new techniques such as fMRI and electroencephalography (EEG). And so, neuroscientific research and technology, by inference, drawn from these findings and increasingly sophisticated technologies, are being applied to legal system rules and processes in the legal field. In this regard, conceptual foundations of neurolaw are raised by the current extant philosophical questions. Theoretical researchers examine the arguments favoring the increased use of neuroscience in law. They ransack the means for assessing its reliability in legal proceedings. Also, theoreticians endeavor to integrate neuroscientific research into substantive legal doctrines. Thus, these effects are covering most aspects of theoretical issues to the practical ones. Maybe the most important books written on the basis of this research method, are as follows: “Minds, Brains, and Law: The Conceptual Foundations of Law and Neuroscience,”^4^ “Neurolaw: Brain and Spinal Cord Injuries,”^27^ “Law, Mind and Brain,”^28^ “Materials on Neurolaw,”^29^ “Law and the Brain”^30^ and “The Neurobiology of Criminal Behavior.”^31^

The major studies in neurolaw theoretical research field are as follows: feasibility of applying neuroscience results in legal system, concept of brain and law, relationship between brain and law, development and technologies of neuroscience in legal system and future, brain disease and disordering legal orders, mental illness and brain injury affection on legal responsibility, right to privacy and brain-imaging, free will on third-party punishment, neuroscience and legal rights, neuroscience and legal freedoms, brain injury citizenship rights, individuals’ right to security towards people with Neurological disease, revolution of legal rules by neurolaw theories.

***Some questions with which neurolawyers are encountering***

Neurolaw attempts to relate the brain to law as well as neuroethics to moral values; so the main question in this branch of neuroscience is how it is and will be used in the legal system? Scientists present a wide variety of possible neuroscience and law intersections.^32^ As it appears, there are a number of distinct ways (including at a minimum in the contexts of Buttressing, Challenging, Detecting, Sorting, Intervening, Explaining, and Predicting) that neuroscience can offer value to law.^33^ In practical sense, evidence suggests that the number of cases involving neuroscientific implications is rapidly increasing. Hereupon, this requires a spacious savvy in both spheres of law and neuroscience. Hence, many questions remain unanswered as to what extent can the brain affect human behavior that would bring about legal effect? and to have fairer and more equitable legal system, what legal rules and precedent should cover this aspect of conducts? How neuroscience should influence criminal and civil law? 

Furthermore, Neurolaw encompasses ethical questions regarding nootropics, more commonly known as mind-enhancing drugs. Nootropics referred to as smart drugs which are memory, neuro, cognitive and intelligence enhancers (supplements, nutraceuticals, and functional foods that improve one or more aspects of mental function, such as working memory, motivation, and attention);^34^ How will these enhancers affect individuals’ legal rights in society? Will it become necessary to use an enhancing drug simply to remain competitive in society? Basically, do people have the right to experiment with substances to modify their own cognition?

With new technologies with which law has confronted, the rise of modern neuroscience expands deeply. It is necessary to be satisfied, on an acceptable level, whether we know enough to draw legally relevant conclusions. Does neuroscience tell us anything we don’t already know from common sense or previous behavioral research? Essentially, are the scientific researchers and medical professionals capable of communicating their ideas in ways accessible for a legal audience? Are there some areas of law to which neuroscience may never be coherent? When we try to have access to the brain information via any Medical science technology, such as fMRI, are we in conflict with the right to privacy? What legal standards could be stated for these problems?

In conceptual sense, the issues are further complicated by the fact that legal doctrine and legal theory make use of our ordinary concepts of mind and mental life. Also, it is extremely difficult to address the relationship among Mind, Brain, and Law. Emphasizing this, neurolaw theorists by utilizing conceptual methodology and philosophical view, focus on the scope and contours being employed in claims involving neuroscience and law. Also, they are relying less on empirical, ethical and practical methods.^20^ The what of the brain, mind, and law, is the main question arises in conceptual, methodological approach; understanding them in the true sense leads us to a mature abstract hindsight of behavior and conducts. Thereby, the path is paved to set of legal rules in order to regulate behaviors in society.

***Primary challenges ahead in Neurolaw***

Most neurolaw findings, besides philosophical, psychological and other related scientific approaches, are mainly based on neuro-medical technology experiments. Corollaries are achieved mostly according to the cognitive study of brain by brain-imaging. In this way, neuroscientists expound neuroscientific data that neurolawyers totally link them to legal effects. An instance is compliant brain-scanning to proving his /her civil or criminal responsibility against the plaintiff who claims it in the tribunal. Based on hermeneutic Interpretation and different perceptions of behavior, the most dreadful problem here is a possible and limited commentary on neuroscientific data and neuro-images.

Neuroscience and law are very different disciplines in nature from laboratory to the courtroom. Discrepancy of language is a critical issue facing neurolaw scientists. It is pretty obvious, neurolawyers are being accosted with many words have slightly varying meanings, or they can be used as a different sense in both sciences. Proof of claim in law must be accurate, reasonable and well-documented. So real problem which arises is probable or almost certain neurological inferences which neurolawyers try to close them to evidence recognized by law;^35^ that is main courtroom problem; this ambiguity of thought extended to state rules or legal processes. All causes a situation in which the cognitive neuroscience influence on the legal field will be so complicated and difficult.

Furthermore, there is an overriding challenge between neuroscience and Human Rights as a branch of Public Law. Neuroscientists strive to get access to neuroscientific data by brain-scanning (such as MRI, FMRI, and EEG), while human rights defenders prevent them. Typically such a quarrel is occurring by assertion of the right to privacy or maybe the right to health; just as conflicting norms between medical law and medical test requirements are originated.

These all lead us to having more concentration on both neuroscience and law to find an appropriate solution and direction between their propositions; this will give origination to neurolaw rules and principles which help both legal and neuroscientific understanding of human behavior. We primarily know: “1- better legal outcomes promote better clinical outcomes for patients with neurological injury; 2- successes in neurolitigation is dependent largely upon the quality and quantity of expert evidence; 3- mutual cooperation among concerned professional enhances the probability of successful neurolitigation; 4- to be successful, clinical and legal professionals require litigation literacy.”^36^.

## Conclusion

The law is not valuable per se. Instead, it is instrumentally used to regulate human behavior due to getting hold of justice; for this purpose we need a comprehensive understanding of legal rules from different scientific standpoints, to be recognized by legal system; one of these most effective sciences which gives hand to law mainly in practical sense, is neuroscience. Neuroscience, exploring brain functions and structures, throws light on the way to better understanding of human behavior. The blend of these two subject-matters (neuroscience and law) has paved the way for neurolaw, in 1990’s. There are two main methods in the neurolaw: theoretical and practical. Until now, most of the neurolawyers have been working on brain functions and neuroscientific data to have a more accurate and fairer justice system, keeping an open staring eye upon successful neurolitigation over several cases in courtrooms. These all highlighted the practical aspect of the subject-matter. However, there were uncertainties about neurolaw but now neurolaw scientists have properly found out that neuroscientific achievements can assist law to have a more reliable decision and rules, and it has shown itself in the field of Procedural law specially civil and criminal responsibility. Of course, neurolaw, while crucial in our legal studies, would help us to apply medical knowledge and technology in the legal area to achieve a more equitable legal system. So to prove liability, to improve the knowledge of the judge with respect to claims, to expand the scope of law, to have a better perception of legal phenomena, even to comprehend the brain and mind to revise the concept of right and many more are windows opened toward our scholarship through neurolaw. It will even associate with Islamic jurisprudence propositions such as those which are discussed in criminal punishments, responsibilities, judicial issues, etc.

## Notes

**How to cite this article: **Petoft A. Neurolaw: A brief introduction. Iran J Neurol 2015; 14(1): 53-8.
